# An unusual renal colic during trans-arterial embolization after trauma and its multidisciplinary management

**DOI:** 10.1093/bjrcr/uaaf039

**Published:** 2025-07-31

**Authors:** Pierpaolo Biondetti, Anna Maria Ierardi, Elisa De Lorenzis, Jacopo Tintori, Emanuele Montanari, Gianpaolo Carrafiello

**Affiliations:** Department of Diagnostic and Interventional Radiology, IRCCS Foundation Cà Granda—Ospedale Maggiore Policlinico, 20122 Milan, Italy; Department of Diagnostic and Interventional Radiology, IRCCS Foundation Cà Granda—Ospedale Maggiore Policlinico, 20122 Milan, Italy; Department of Urology, IRCCS Foundation Cà Granda—Ospedale Maggiore Policlinico, 20122 Milan, Italy; Department of Clinical Sciences and Community Health, University of Milan, 20122 Milan, Italy; Postgraduation School in Radiodiagnostics, University of Milan, 20122 Milan, Italy; Department of Urology, IRCCS Foundation Cà Granda—Ospedale Maggiore Policlinico, 20122 Milan, Italy; Department of Clinical Sciences and Community Health, University of Milan, 20122 Milan, Italy; Department of Diagnostic and Interventional Radiology, IRCCS Foundation Cà Granda—Ospedale Maggiore Policlinico, 20122 Milan, Italy; Department of Oncology and Oncohematology, University of Milan, 20122 Milan, Italy

**Keywords:** interventional radiology, embolization, NBCA, glubran, bleeding, migration, complication

## Abstract

Selective embolization is the treatment of choice for traumatic renal pseudoaneurysm (PSA) in stable patients. N-Butyl-2-cyanoacrilate (NBCA) is an embolic agent frequently used to embolize peripheral lesions. N-Butyl-2-cyanoacrilate is one of the most widely used embolic materials because it is easy to prepare, it acts quickly and is highly cost-effective. Its use, however, requires a learning curve before becoming confident and being able to handle it safely. We describe a case of embolization of a renal traumatic PSA without clear pre-procedural CT-evidence of artero-calyx fistula in which the migration of NBCA in the renal pelvis occurred during the procedure. We report the successful multidisciplinary management of this complication.

## Introduction

Arterial blush, pseudoaneurysm, artero-venous fistulas, artero-caliceal, and arterio-ureteral fistulas may occur after either blunt or penetrating renal trauma or iatrogenic injury.^1^ Treatment depends on the hemodynamic status, with nonoperative management being the first choice in stable patients, including embolization.^2^ There are no guidelines on the embolic agent to be used with the decision depending on the operator’s experience, confidence, and knowledge.

Commonly used embolic agents include coils, plugs, or liquid agents such as n-butyl-2-cyanoacrylate (NBCA) and ethylene vinyl alcohol copolymers (EVOH).^3^

n-Butyl-2-cyanoacrylate, also called glue, has proved its safety and efficacy in different pathologies and districts,^3^^,^^4^ but is more difficult to control than other agents, requiring technical knowhow to be used.

Reported complications include nontarget embolization, ischemia, and microcatheter’s entrapment.^5^^,^^6^

We present an unusual case of NBCA nontarget embolization occurred during endovascular embolization for the treatment of a renal traumatic arterial lesion.

## Case report

**Figure 1.**
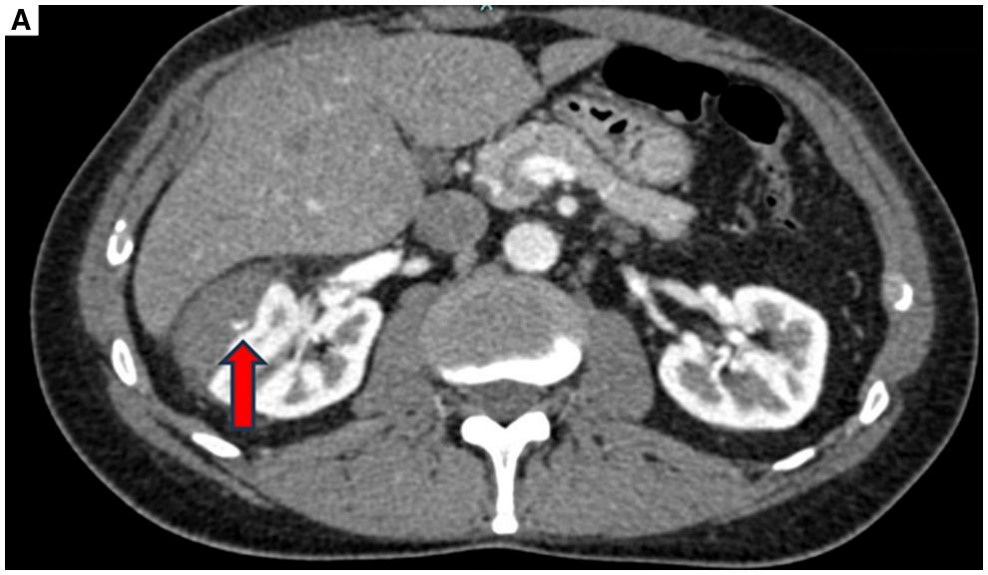
Active bleeding. (A) Axial CT image in the arterial phase demonstrating a right renal hematoma with active bleeding (arrow). Abbreviation: CT = computed tomography.

A 45-year-old man was transported to our institution after a bicycle accident with blunt trauma to his right flank. At presentation, he was stable with the following vitals: HR 68 bpm; BP 135/75 mmHg; Hb 13.7 mg/dL. The patient did not report gross hematuria, but urine analysis showed microhematuria. Contrast-enhanced CT (CECT) revealed a right renal hematoma with active bleeding (Figure 1) classified as Grade III trauma lesion according to AAST renal injury grading scale.^2^

**Figure 2.**
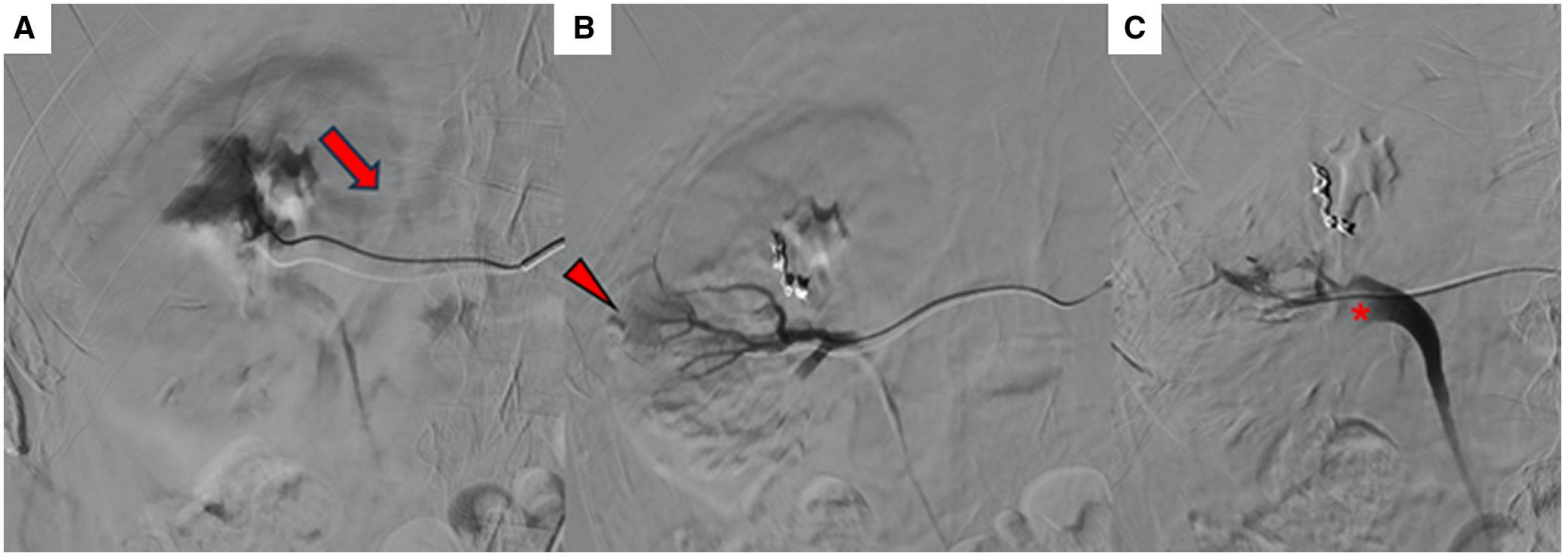
(A) Angiography performed after super-selective catheterization, demonstrating the presence of an arterio-venous fistula (arrow) later embolized with coils. (B) Angiographic image after microcatheterization of another arterial renal vessel shows the presence of a focal vascular injury (PSA, void arrow), which was embolized using NBCA and Lipiodol. (C) Angiographic control after embolization reveals an arterio-caliceal fistula, with a filling defect in the renal pelvis (asterisk). Abbreviations: NBCA = n-butyl-2-cyanoacrylate; PSA = pseudoaneurism.

A multidisciplinary indication for embolization was given. Renal angiography confirmed a small peripheral arterial blush and a small artero-venous fistula (Figure 2). The latter was embolized using a 2.7 F Progreat microcatheter (Terumo Medical, Japan), and 2-3mm microcoils (Azur, Terumo Medical, Japan). The feeder to the arterial blush was then reached and embolized with a 1:1 mixture of NBCA (Glubran 2; GEM, Italy) and Lipiodol (Guerbet, France) injected in a controlled fashion and under fluoroscopic control.

During the injection, an extravasation of less than 0.1 ml of the NBCA mixture from the artery into the calyx and then into the pelvis was observed, and the patient suddenly referred right flank colic pain (VAS score: 8/10). Fluoroscopy showed a polymerized NBCA conglomerate inside the renal pelvis of approximately 3 mm, mimicking a stone. It is important to note that the extravasation was not immediately noted and was not acknowledged until the patient started having pain.

The pain was responsive to the i.v. administration of Ketorolac 30 mg and ½ vial of Fentanyl-hameln 50 μg/ml solution (VAS score 4/10).

**Figure 3.**
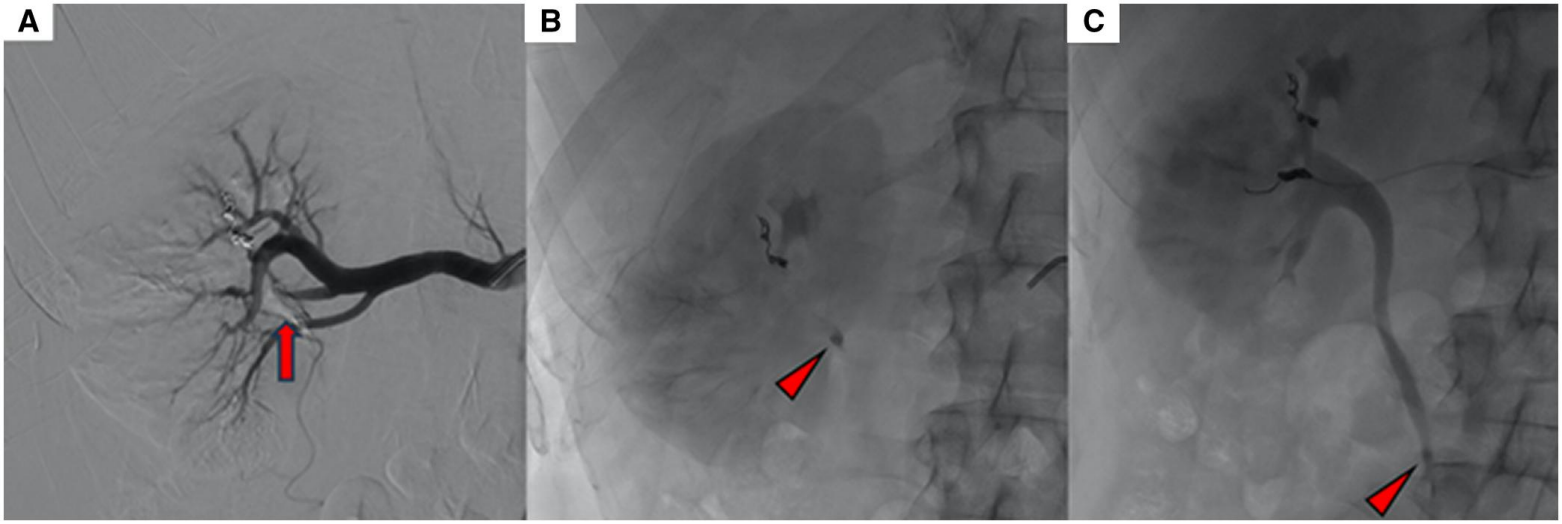
The NBCA conglomerate. (A) Repeated angiography shows NBCA cast inside the renal pelvis (arrow). (B) Subsequent fluoroscopic images show the NBCA conglomerate at the ureteropelvic junction and later in the middle portion of the ureter. (C) Causing partial occlusion (arrowhead). Abbreviation: NBCA = n-butyl-2-cyanoacrylate.

A subsequent angiogram confirmed the presence of an artero-caliceal fistula. The procedure was completed by embolizing the fistula with a 4 mm coil. Fluoroscopy showed a slow migration of the radiopaque NBCA conglomerate through the ureter (Figure 3), which reached the middle portion after about 30 minutes. A decision was made to wait 24 hours to allow the conglomerate to eventually reach the bladder and be eliminated.

**Figure 4.**
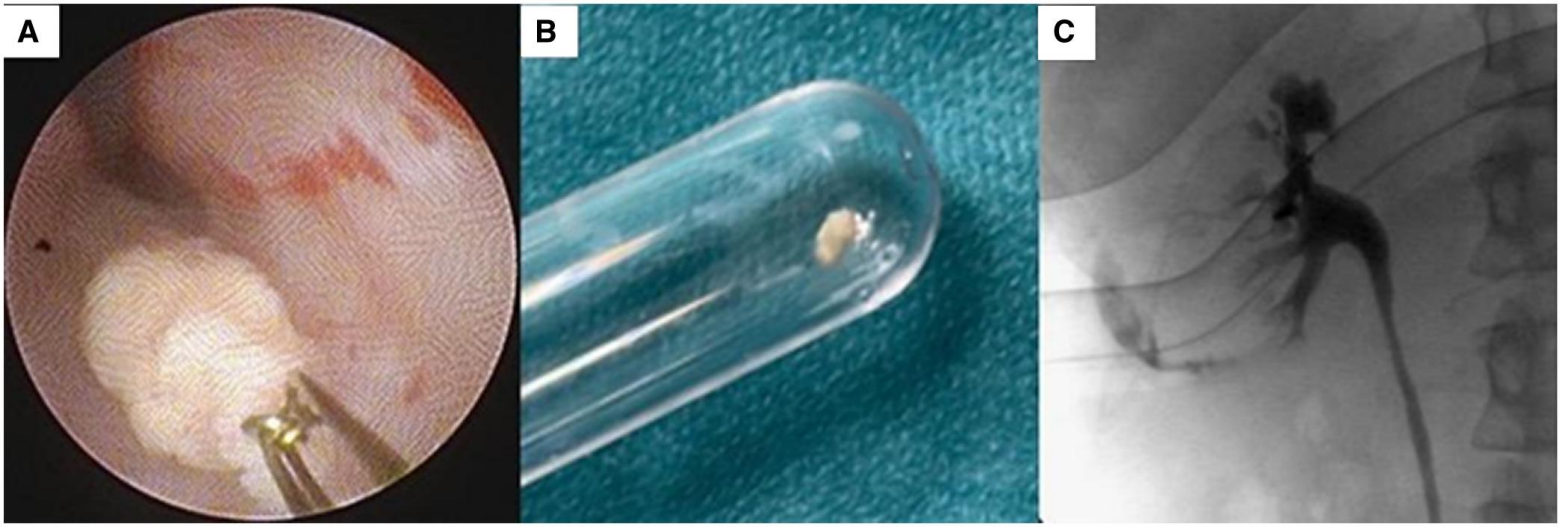
Urology procedure. (A) Endoscopic view of the extraction of the NBCA conglomerate. (B) The NBCA conglomerate extracted. (C) The final pyelography showing an extravasation of contrast medium at the level of the inferior right calyx. Abbreviation: NBCA = n-butyl-2-cyanoacrylate.

The following day, an unenhanced CT showed persistence of the NBCA conglomerate inside the pelvic ureter and a mild dilation of the ipsilateral excretory system; VAS score was 5/10 despite antalgic therapy. At this point, urological intervention was required to extract the conglomerate. After placing a safety hydrophilic guidewire, a semirigid ureteroscopy with gravity irrigation was performed which showed the foreign body made of NBCA inside the distal ureter, which was extracted using a 2,2 ch Nitinol basket (Coloplast). Ureteroscopy was conducted up to the renal pelvis and was negative for residuals, with no ureteral lesions identified. The retrograde pyelography demonstrated an extravasation of contrast medium at the level of the inferior right calyx (Figure 4) that was managed with insertion of a JJ ureteral stent 6 ch × 28 cm, which was kept for 10 days, and a 5 day therapy with i.v. ceftriaxone 2 g.

Two weeks later, the patient presented to the emergency department with high fever and right flank pain. Laboratory exams were as follows: creatinine 1.15 mg/dl, CRP 2.06 mg/dl, WBC 13 610/mmc, Hb 12.4 mg/dl. Empiric antibiotic therapy was started with piperacillin/tazobactam. A new CECT scan showed pyelonephritic foci in the right kidney and stability of the hematoma. After positivity of blood cultures for *Staphylococcus aureus*, antibiotic therapy was shifted to i.v. Oxacilline. After 2 weeks of outpatient parenteral antimicrobial therapy the JJ stent was removed. At the 2 months follow-up, the patient was asymptomatic, laboratory exams showed normal values of creatinine, Hb and inflammatory markers. United States demonstrated a slight size reduction of the right renal hematoma with no hydronephrosis.

## Discussion

n-Butyl-2-cyanoacrylate is appreciated for embolization due to its rapid polymerization and effective occlusion of vessels. However, unexpected migration into the urinary tract can have significant consequences, including symptomatic urinary obstruction, infection and impaired renal function.

In the first case of NBCA urinary migration reported, endoscopic and laparoscopic treatment attempts were not successful, and the foreign body was later spontaneously passed out by chance.^7^ Other cases were successfully managed with ureteroscopic procedures, including simple retrieval of the foreign body^8^ or initial laser lithotripsy followed by fragment removal;^9^ therefore, confirming that treating the NBCA conglomerate like any normal kidney stone might be a possible recommended approach when this specific complication arises. In these cases, there were no long-term consequences for renal function. A case similar to the present but involving EVOH occurred to us in the past,^1^ with some interesting differences. In the current case symptoms, mimicking a renal colic developed immediately, while with EVOH, the patient remained asymptomatic. This might be explained by the shape acquired by the polymerizing agent: EVOH formed multiple tiny speckles while NBCA aggregated as a single conglomerate, resembling a kidney stone, probably because of immediate polimerization. Moreover, while EVOH autonomously passed into the bladder and was retrieved by the IR team with a goose-neck catheter, the present case required urological intervention.

Several factors can contribute to the migration of embolic agents. These include the amount and rate of NBCA injection, the lipiodol/NBCA mixture ratio, the pressure and flow dynamics within the renal vasculature, and the presence of unrecognized traumatic fistulas between the vascular and the urinary tract.

## Conclusion

This case underlines the need for maximum attention in identifying any source of unexpected complication during the procedure, especially when using liquid embolics; the fistula did not reveal its presence until some degree of embolization was performed.

It also highlights the value of a multidisciplinary approach, which is the way to obtain the best results; bleeding was stopped acutely and urinary complications had no long-term sequelae thanks to urological intervention.

## Learning points/take home messages

- This case underlines the need for maximum attention in identifying any source of unexpected complication during the procedure, especially when using liquid embolic agents.
- The case shows how some endovascular complications can be challenging to detect: the fistula did not reveal its presence until some degree of embolization was performed.
- The case highlights the importance of multidisciplinary management, especially in unusual cases.

## References

[1] Ierardi AM , Pesapane F, Arrichiello A, Fontana F, Piacentino F, Carrafiello G. Migration of ethylene vinyl alcohol copolymer in the urinary tract successfully managed. Medicina (Kaunas, Lithuania). 2019;55:234. 10.3390/medicina55060234 31159307 PMC6630371

[2] Coccolini F , Moore EE, Kluger Y, et al; WSES-AAST Expert Panel. Kidney and uro-trauma: WSES-AAST guidelines. World J Emerg Surg. 2019;14:54. 10.1186/s13017-019-0274-x 31827593 PMC6886230

[3] Chakraverty S , Flood K, Kessel D, et al CIRSE guidelines: quality improvement guidelines for endovascular treatment of traumatic hemorrhage. Cardiovasc Intervent Radiol. 2012;35:472-482. 10.1007/s00270-012-0339-7 22271075

[4] Chun JY , de Haan M, Maleux G, Osman A, Cannavale A, Morgan R. CIRSE standards of practice on management of endoleaks following endovascular aneurysm repair. Cardiovasc Intervent Radiol. 2024;47:161-176. 10.1007/s00270-023-03629-1 38216742 PMC10844414

[5] Loh Y , Duckwiler GRO, Trial I. A prospective, multicenter, randomized trial of the onyx liquid embolic system and N-butyl cyanoacrylate embolization of cerebral arteriovenous malformations. J Neurosurg. 2010;113:733-741. 10.3171/2010.3.JNS09370. 20433277

[6] Takeuchi Y , Morishita H, Sato Y, et al; Committee of Practice Guidelines of the Japanese Society of Interventional Radiology. Guidelines for the use of NBCA in vascular embolization devised by the committee of practice guidelines of the japanese society of interventional radiology (CSIR), 2012 edition. Jpn J Radiol. 2014;32:500-517. 10.1007/s11604-014-0328-7. 24889662

[7] Chen WJ , Wang SC, Chen SL, Kao YL. Foreign body in the ureter: a particle of glue after transarterial embolization of a renal pseudoaneurysm during percutaneous nephrostomy. J Chin Med Assoc. 2012;75:183-186. 10.1016/j.jcma.2012.02.011 22541148

[8] Prasad SK , Agrawal O, Mittal A, Panwar VK, Dubey D. Cyanoacrylate glue masquerading as an obstructive calculus: rare sequelae of angioembolization for renal pseudoaneurysm. Cureus. 2023;15:e35135. 10.7759/cureus.35135 36949971 PMC10026603

[9] Inchingolo R , Antonucci M, Pinto F, Cina A. Proximal ureteric obstruction caused by glue migration following selective renal artery embolization. J Vasc Intervent Radiol. 2015;26:448-450. 10.1016/j.jvir.2014.11.016 25735529

